# Naringenin-loaded nanoparticles modulate HIF-driven oxygen-sensing pathways in lung adenocarcinoma cells

**DOI:** 10.1186/s13104-025-07133-2

**Published:** 2025-02-12

**Authors:** Eman M. Ragab, Doaa M. El Gamal, Tarek M. Mohamed, Abeer A. Khamis

**Affiliations:** Biochemistry Division, Chemistry Department, Faculty of Science, Tanta University, Tanta, 31527 Egypt

**Keywords:** Naringenin, Hypoxia-inducible factor, Chitosan, Hypoxia, Lung cancer

## Abstract

**Background:**

Hypoxia is a common symptom of lung cancer. Proliferation and neovascularization mediated by hypoxia-inducible factors (HIF) influence several adaptations. It has recently been established that naringenin (NAR) and its nanoparticles are chemo-preventive flavonoids in lung cancer.

**Aim:**

Adjust HIF activity by reviving oxygen-sensing enzyme activity while considering possible therapeutic targets.

**Method:**

The bindings of NAR to target proteins were examined using computational modeling techniques. Additionally, NAR nanoparticles (NARNPs) were synthesized and characterized. Normal fibroblast cells and A549 cells were used to determine cytotoxicity. Colorimetric analysis of α-ketoglutarate detection for hydroxylases.

**Results:**

According to molecular modeling, NAR and target proteins have a high affinity. The PHD and FIH activities in A549 are significantly stimulated.

**Conclusion:**

NAR and NARNPs diminish hypoxia in lung cancer by stimulating oxygen-sensing hydroxylases.

**Supplementary Information:**

The online version contains supplementary material available at 10.1186/s13104-025-07133-2.

## Introduction

Hypoxia, or the lack of oxygen, is a characteristic of lung tumors that encourage genomic instability, increased aggressiveness, and metastasis. One of these causes is the disruption of the normal oxygen-sensing mechanisms, which causes cells to respond as though there is insufficient oxygen [1]. Hypoxia-inducible factors (HIFs), transcription factors whose levels are (typically) extremely sensitive to changes in O_2_ tension, are important cellular regulators of chronic O_2_ homeostasis [2]. HIF is a heterodimeric transcription factor, and in human cells, the amount of the HIF-1α domain is modulated by enzymes called HIF hydroxylases [3]. The oxygen-dependent degradation domains (ODDs) at the N- and C-termini of HIF-1, Pro 402, and Pro 564 are respectively hydroxylated by the prolyl hydroxylase (PHDs) under normal oxygen circumstances. Due to these changes, the von Hippel-Lindau tumor suppressor protein (VHL) interacts more favorably with other proteins for ubiquitination and proteasome-mediated destruction [4]. On the other hand, Asn803 in the CAD (C-terminal activation domain) of HIF-1 undergoes asparagine hydroxylation, which is catalyzed by factor inhibiting hypoxia (FIH), preventing interaction between HIF- and the co-transcriptional activator, p300 [5]. HIF hydroxylase activity decreases, HIF-α levels rise, and it can dimerize with HIF-β, to increase the activity of genes implicated in the response to hypoxia. Consequently, the identification of a novel drug that specifically targets the HIF-1-regulated pathway represents a promising chemotherapeutic approach for human lung cancer treatment. Naringenin (NAR), a common flavonoid, has been proposed as an antioxidant as well as having anti-cancer characteristics [6]. It has been demonstrated to have an anti-carcinogenic impact on lung cancer cells by altering the processes of cell proliferation, apoptosis, and metastasis because of its antioxidant activity [7, 8]. However, the hydrophobic and crystalline structures are primarily responsible for its instability, limited oral bioavailability, and water solubility. Additionally, it lacks targeting and is susceptible to rapid breakdown in an acidic environment. These disadvantages make it difficult to use it medicinally effectively [7]. Nevertheless, encapsulation NAR helps to shield them from metabolic activity, to encourage controlled and prolonged drug release, and to improve their bioavailability. Chitosan (CS) is utilized to encapsulate the flavonoid NAR due to its unique properties [9]. A naturally occurring polymer CS is produced when chitin undergoes partial N-deacetylation [10]. Because of its special qualities such as hydrophilicity, non-toxicity, biodegradability, biocompatibility, and affordability, it is employed as a nanocarrier. It can interact with different epithelia and the potential for cohesion. Additionally, the CS-NPs shield the drugs from outside influences like pH and enzymes [10]. Because they have increased surface area to absorb, bind, and transport the medicines, nano encapsulations of CS polymers are efficient drug delivery vehicles. By encouraging the ionic interaction of CS amino groups with their anionic groups, tripolyphosphate (TPP) can be used as a cross-linker and raise the solution’s ionic strength [11]. Ionic cross-linking with TPP resulted in CS-NPs that demonstrated improved drug loading efficiency and a longer drug release duration. Consequently, our studies aim to target and investigate the effect of HIF-1-regulated hydroxylases by loading NAR on CSNPs as drug carriers as it’s a promising chemotherapeutic approach for the treatment of hypoxia in lung cancer.

## Methods

### In silico studies & Drug likeness/pharmacophore and ADMET profile analysis

The interaction of NAR with proteins involved in the HIF-regulated pathway proteins was studied by molecular docking through the methodology proposed in [12].

### Nanoparticles preparation

CS-NPs were prepared by the ionic gelation technique. Briefly, a concentration of 0.5 mg/mL of NAR was added to CS to TPP ratios of 5:1 (w/w), which were then agitated to achieve complete dissolution at pH 4.8 [7].

#### Characterization ofs NARNP

A UV-Vis 2550 spectrometer (Shimadzu) [13], Fourier transform infrared (FTIR) spectroscopy (JASCO, FT/IR-6100) [14], XRD analysis (GNR, APD2000PRO, Italy) [15]. A transmission electronic microscope (TEM) (JEOL-2100) [16], SEM connected to a JEOL JSM-6510 LV energy dispersive spectroscopy (EDS) device [17]. Zetasizer Nano Series instrument (Horiba Nano partica SZ-100, Japan), the Zeta potential (ζ) of the mixture was determined [18]. The release study of NAR from NARNPs was made using formula at 37 °C and in 1X PBS pH 7 [7].

### Cell viability and cell proliferation assays

Briefly, on 96-well plates, 2 × 10^3^ human pulmonary adenocarcinoma A549 cells (# ATCC CCL-185) and normal lung fibroblast cells WI-38 (# *ATCC WI-38*)/samples were planted, and after 48 h, they were exposed to varying doses of NAR, NARNPs, and 5-FLU. As described [19].

### Prolyl hydroxylase (PHD) & asparagine hydroxylase (FIH) activity assays

Briefly, five mature male Swiss albino mice weighing between 28 and 30 g were acquired from the Faculty of Pharmacy, Alexandria University, in Egypt. The mice were kept in an appropriate temperature range with a 12-hour light/dark cycle. Following intraperitoneal (*i.p.*) administration of sodium pentobarbital anesthesia (300 mg/kg) [20]. The mice were cervically dislocated to finish the experiment. From the peritoneal cavity, the liver was extracted, and the gallbladder was then found and extracted using a scalpel. Moreover, the cytosol fraction was isolated from 60 × 10^6^ for A549 and normal cells in accordance using the previously mentioned procedure [14].The Guidelines for the Care and Use of Laboratory Animals published by the National Institutes of Health were adhered to in the care of the experimental animals. The Egyptian Ethical Committee of Tanta University, Faculty of Science **(#IACUC-SCI-TU-0287)** oversaw the animal studies. Time-course assays were carried out in the absence/presence of IC_50_ of NAR, NARNPs, and 5-FLU. Initially, 100µL of 5mM α-ketoglutarate, 100µL (500 Mm) HEPES, 100µL (20 mM) Dithiothreitol, 100µL 1.5 Mm FeSO_4_, 100µL (5mM) ascorbic acid, 100µL (5mM) proline for PHD or 5mM Aspartic acid for FIH activities were added to prepare the in vitro hydroxylation assay [19].

## Results

### Molecular docking

Auto Dock Vina was believed to be the primary virtual screening platform. As shown in Fig. 2, the binding affinity of NAR with the targets PHD, FIH, and VHL was − 10.1 kcal/mol, − 8.1 kcal/mol, and − 8.3 kcal/mol, in that order (Table S4, Fig. 2). Furthermore, NAR was shown to have good drug-likeness qualities, target precision, and safety for human use [21].

**Fig. 1:**
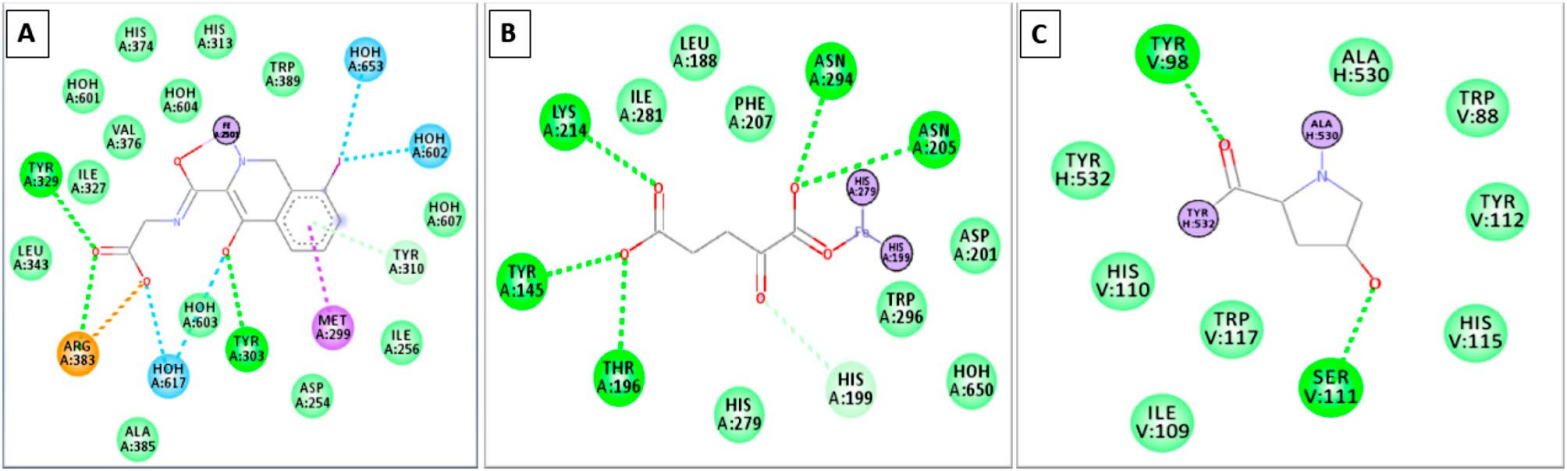
2D representations of the NAR and active pocket interaction **(A)** PHD **(B)** FIH **(C)** VHL-like protein’s active site at left and right sides respectively

### Naringenin-loaded nanoparticles characterization results (NARNPs)

**Fig. 2:**
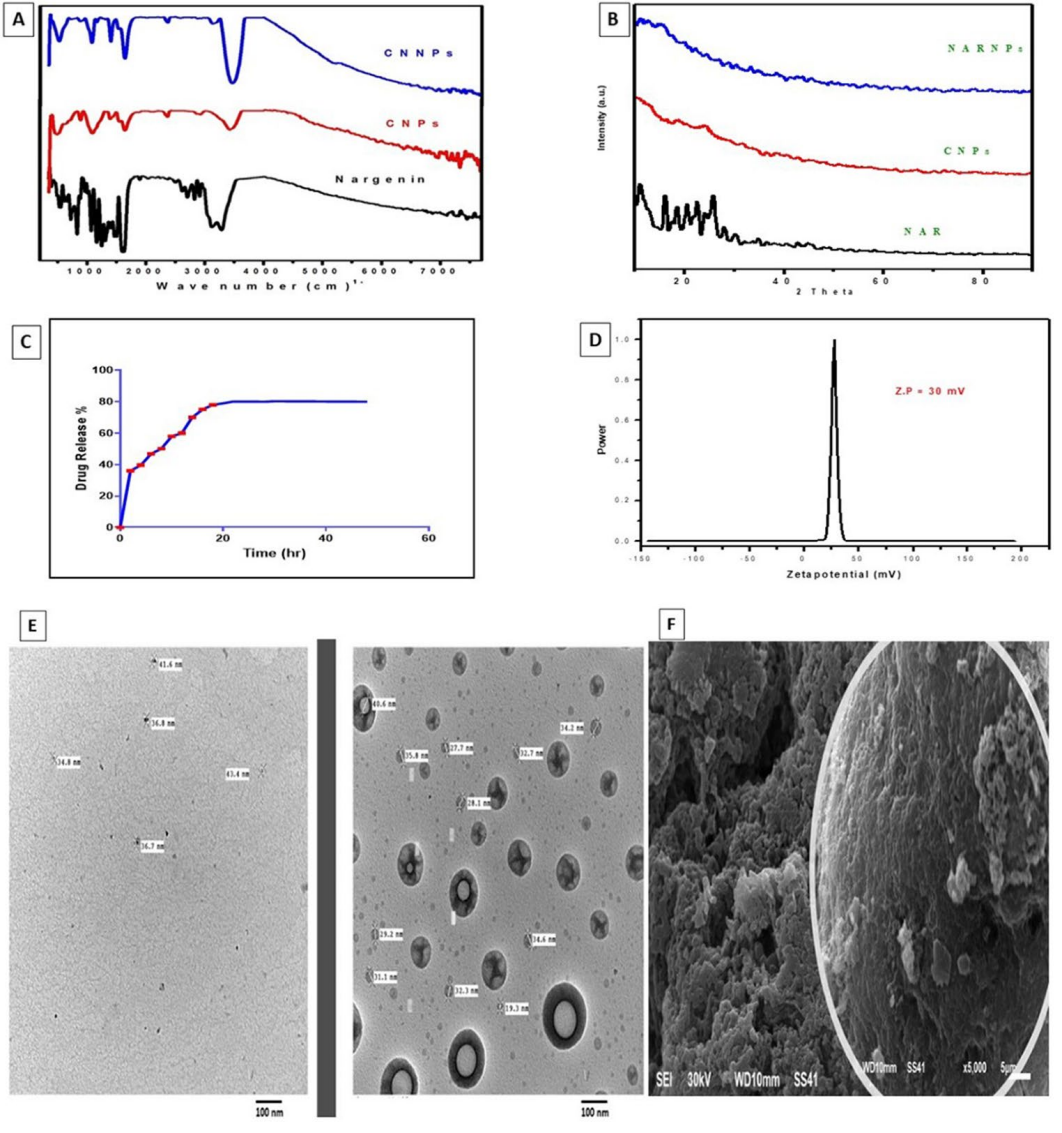
Illustrates nanoparticles characterization **(A)** Fourier transform - infrared spectroscopy of NARNPs, **(B)** The XRD pattern of NARNPs, NAR, and CS nanoparticles, in that sequence **(C)** The in vitro release profile of NAR from the NARNPs, **(D)** The zeta potential of NAR nanoparticles, **(E)** TEM micrograph displaying chitosan nanoparticles (CNPs) on the left side with a size range of 30–40 nm and NARNPs on the right side with a size range of 19–40 nm, **(F)** The surface structure of NAR-chitosan nanoparticles

Over time, the NAR release profile from NARNPs in vitro was evaluated. As demonstrated, 80% of NAR released from NARNPs in 20 h in Fig. 2A. Positive zeta potential is frequently seen in chitosan nanoparticles. The total positive zeta potential of our formulations varied from + 30 mV to + 50 mV as in Fig. 2B. A characteristic absorption band that appears between 3,310 and 3,120 cm^− 1^ in the case of NAR alone Fig. 2C. NAR demonstrated the crystalline nature of the material in an X-ray diffractogram. The amorphous nature of chitosan nanoparticles was revealed in Fig. 2D. The form and size of nanoparticles varied between 19 and 40 nm, with a spherical shape and a smooth surface Fig. 2E. SEM analysis was used to evaluate the size, surface morphology, and homogeneity of NARNPs as demonstrated in Fig. 2F.

### Cell viability

The MTT method was used to measure cell growth. Next, the results were contrasted with those attained with 5-FLU acting as a positive control. There was a substantial (*p ≤ 0.01*) drop in the viability cells as the treatment concentration increased. The IC_50_ values of NAR, NARNPs, CNPs, and 5-FLU in A549 cells were 61, 35.2, 394, and 5.5 µg/mL respectively, whereas the IC_50_ values of these compounds were 461, 71, 311, and 8.07 µg/mL respectively in normal fibroblasts.(Table S1).

### PHD & FIH activities

**Fig. 3:**
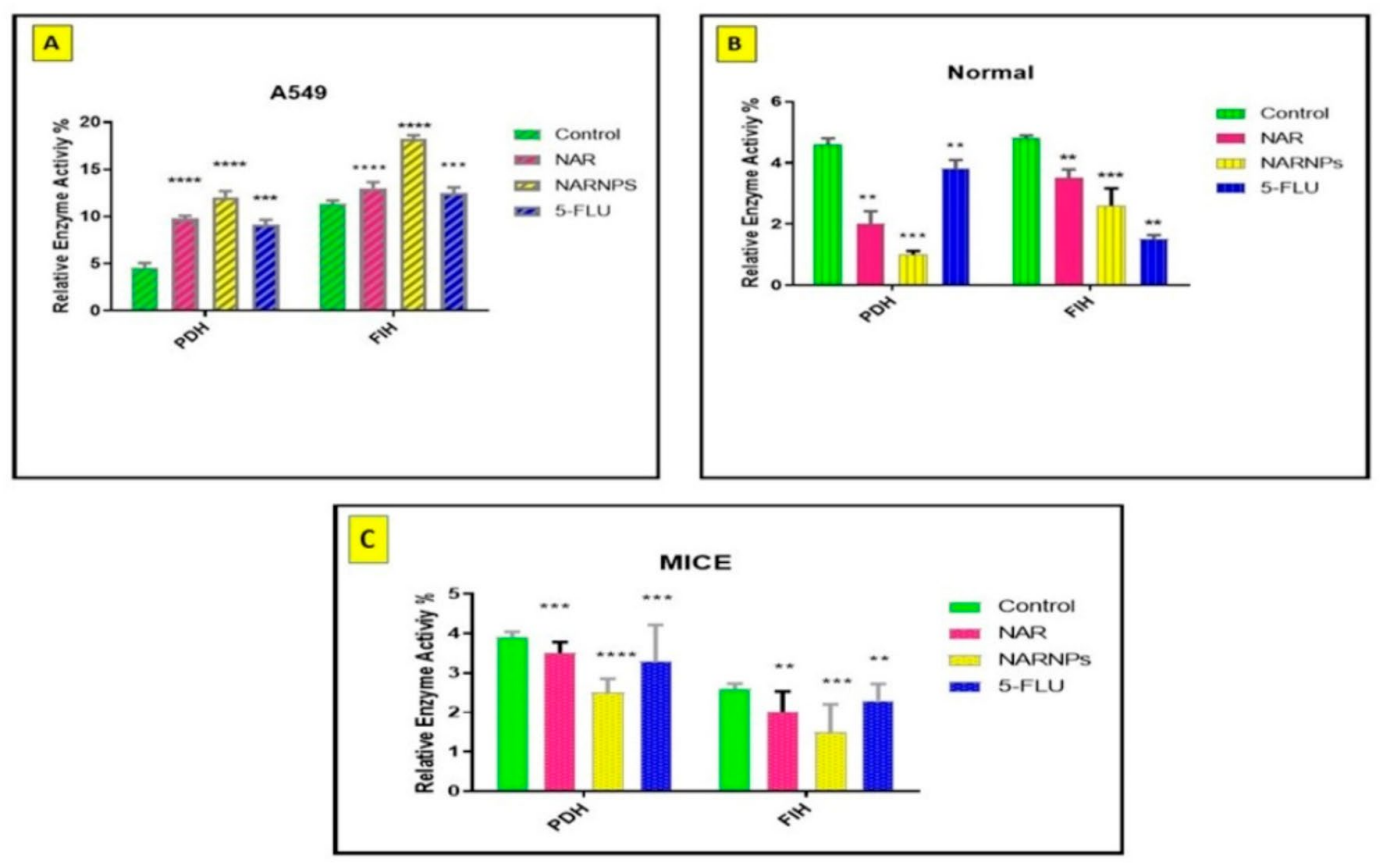
The influences of NAR, NARNPs, and 5-FLU on PHD and FIH activities **(A)** Relative activities are upsurged in sequence (NARNPs > NAR > 5-FLU) in A549 for both PHD and FIH. **(B)** Normal, there was significant fluctuation for both PHD and FIH (5-FLU > NAR > NARNPs) (NAR > NARNPs > 5- FLU), respectively (**C)** Normal mice, (NAR = 5-FLU > NARNPs) towards activities. Where ****p ≤ 0.0001, ***P ≤ 0.001, **P ≤ 0.01, *P ≤ 0.05, # for non- significant against control and each other

NAR, NARNPs, and 5-FLU significantly stimulate PHD and FIH activities in A549 cells with sequence (NARNPs > NAR > 5-FLU) as shown in (Table S2, Fig. 3A). On the other hand, there is a slight decrease in both activities in normal cells and mice as in (Table S3, Fig. 3B, C).

## Discussion

Hypoxia is the main hallmark of lung cancer which is the primary cause of cancer-related deaths globally and is still a difficult public health concern [22]. Due to its pivotal function in the adaptive reaction to hypoxia, the oxygen-sensing pathway has garnered significant attention as a potential therapeutic target for lung cancer. We can forecast the binding behavior and affinity between NAR and our target proteins PDH, FIH, and VHL that may occur during the non-covalent binding process by molecular docking, Moreover, the NAR-PDH, FIH, and VHL proteins respective Gibbs free energy values of -10.1, -8.1, and − 8.3 kcal/mol were determined, confirming their high affinity and spontaneous complex formation [23]. It has been demonstrated that NAR possesses good drug-likeness properties, target accuracy, and human safety [21]. To prepare CS-NPs, TPP was cross-linked ionically at a ratio of 1 to 2.5. While Kumar, Birundha, et al. (2015) showed that a CS: TPP ratio at 5:1 in 0.5 mg/ml of NAR concentration displayed a significantly higher percentage of encapsulation efficiency (~ 70%), our formulation with a 1:2.5 CS: TPP ratio at 0.5 mg/ml by ~ 96%. The surface charge of the particles, as measured by zeta potential, our formulation agreed with other previous studies which increased from + 22.73 mV to + 58.43 mV [7]. High positive charge particles can penetrate tissues and adhere to mucous membranes, making them more stable. In terms of drug release our formulation showed 80% NAR release from NARNPs in 20 h. In prior studies, depending on the mass ratio, the release of entrapped NAR from CS-NPs was found to be between 45 and 55% [24, 25]. The molecular interactions of the CS-NPs with NAR were obtained from FT-IR suggesting that hydrogen bond interactions linked the NAR to the CS-NPs [7, 26]. We confirmed the structure by using an X-ray diffractogram, and NAR showed that the material was crystalline. It was discovered that chitosan nanoparticles are amorphous. It showed a prominent peak at 22°. Our nanoparticles had a smooth surface, spherical shape, and varied in size from 19 to 40 nm. The size of NARNPs, surface shape, and homogeneity were assessed by SEM analysis with a ratio of 1:2.5. This result was contrary to previous studies were found that the average diameter of 5:1 ratio CS-NPs was 53.2 nm with an average diameter of 407.47 nm, the encapsulated NAR in CS-NPs (CS-NPs/NAR) revealed spherical-shaped NPs [7]. It has been observed that when NAR is encapsulated, its characteristics are preserved. The cytotoxicity impact in A549 cells indicated that free and nano formulations of NAR had cytotoxic effects on cancer cells while normal had no cytotoxicity this study agreed with the prior study [27]. PDH and FIH activities in A549 cells are considerably stimulated by NAR, NARNPs, and 5-FLU in the order (NARNPs > NAR > 5-FLU), respectively. The quantity of 2-OG that is still present in the reaction mixture has a direct relationship with the intensity [19]. These results were supported by previous studies that indicated flavonoids decreased the stability of HIF-1α, with enhanced prolyl hydroxylation, which causes it to be more susceptible to ubiquitination and destruction [8, 28]. In contrast to the inhibition of HIF prolyl hydroxylases (PHDs), there has been less research on the activators of FIH [29]. For the first time, our data support theoretically strong affinity for FIH active site with binding affinity − 8.1, and practically the relative activity was 18.2%, 13%, and 12.4% for NARNPs, NAR, and 5-FLU, respectively in A549.

### Limitations

Scientific studies suggest that the likelihood of serious side effects occurring is low and that its profile should be safer than that of other chemotherapy drugs. We predicted that NAR-CSNPs will be a promising activator to lessen hypoxia in lung cancers for the foreseeable future. Additionally, chitosan nanoparticles coated with NAR are opening the door for the development of innovative hypoxic lung cancer therapies. However, further studies have needed for genetic and epigenetic investigations in vivo and in vitro are required to be comprehended in regulated HIF pathways.

## Conclusion

Lung cancer was estimated as a hypoxic state which obligates the cell to tumorigenesis. Therefore, Oxygen-sensing prolyl hydroxylase (PHD) is responsible for proteasomal degradation, and factor inhibiting hypoxia (FIH) for transcription activity could be a therapeutic target for hypoxia in lung cancer. Our recent studies were done on the effect of NAR and its nanoparticles theoretically and practically on PHD, FIH. Our molecular modeling showed a strong affinity between NAR and target proteins. Further enzymatic activity assays showed an upsurge in PHD and FIH hydroxylase enzymes. We conclude that NAR and NARNPs efficiently alleviate oxygen-sensing HIF hydroxylases through the hypoxic pathway in lung cancer.

## Electronic supplementary material

Below is the link to the electronic supplementary material.

Supplementary Material 1

## Data Availability

All data generated or analysed during this study are included in this published article [and its supplementary information files].

## Abbreviations

HIF1: Hypoxia-Inducible Factor 1
ODD: Oxygen-dependent degradation domains
CAD: C-terminal activation domain
PHDs: Prolyl hydroxylase
VHL: Von Hippel-Lindau tumor suppressor protein
FIH: Factor Inhibiting Hypoxia
NAR: Naringenin
NARNPs: Naringenin Nanoparticles
5-FLU: 5-Fluorouracil
