# Supplementary material for: Naringenin-loaded nanoparticles modulate HIF-driven oxygen-sensing pathways in lung adenocarcinoma cells
Source: BMC Res Notes. 2025 Feb 12;18:64. doi: 10.1186/s13104-025-07133-2 (PMC11817823; doi:10.1186/s13104-025-07133-2)
Supplement: Supplementary file 1 — Supplementary Material 1 [file 13104_2025_7133_MOESM1_ESM.docx]

**Materials**

Naringenin (NAR) (>99%), chitosan (CS) (Mw = 100–300 kDa, 70–75% deacylated), Trisodium Penta polyphosphate (TPP, 99%), Tween 80, cytosolic extraction buffers and MTT [3–(4,5-dimethylthiazol-2-yl)-2,5-diphenyltetrazolium bromide]. These analytical-grade compounds were all purchased from the German company Sigma-Aldrich.

**Statistical analysis**

At least three replications of each experiment were conducted. The mean ± SEM is used to present the data. When appropriate, the t-test or a two-way ANOVA was used for statistical comparisons, (GraphPad 8 Software Inc., USA).  *P* values less than 0.05 were regarded as statistically significant.

**Table 1.** IC_50_ for 48 hours of 5-FLU, NARNPs, CNPs, and NAR.

| Ic_50_ Concentration of treatment  (μg/mL) | Normal cells | A549 cells |
| --- | --- | --- |
| Negative control  (Untreated cells) | 100 ± 0 | 100 ± 0 |
| NAR mean± SEM | 461.6 ± 0.49 | 61 ± 0.23 |
| NARNPs mean± SEM | 71 ± 0.0849 | 35.2 ± 0.014 |
| CNPs mean± SEM. | 311.1 ± 0.013 | 394± 0. 05 |
| 5-FLU mean± SEM. | 8.07 ± *0.025* | 5.5± 0.02 |

**Table 2.** Influence of NAR, NARNPs, and 5-FLU on PHD activity in, A549 cells, normal cells, and mice.

| Mice | Normal | A549 | Activity |
| --- | --- | --- | --- |
| 3.9± 0.14 | 5± 0.2 | 4.57± 0.5 | **Untreated**  **mean± SEM.** |
| 3.5± 0.28 | 4.5± 0.4 | 9.77 ± 0.32 | **NAR** |
| 3.1± 0.2 | 4.2 ± 0.12 | 12.05 ± 0.03 | **NARNPs** |
| 3.3 ± 0.1 | 4.7 ± 0.4 | 9.1 ± 0.5 | **5-FLU** |

**Table 3.** Impact of NAR, NARNPs, and 5-FLU on FIH activity in A549, normal cell, and mice

| Mice | Normal | A549 | Activity |
| --- | --- | --- | --- |
| 2.5 ± 0.13 | 4.8± 0.1 | 11.35± 0.3 | **Untreated**  **mean± SEM.** |
| 2.2± 0.53 | 4.5± 0.2 | 13 ± 0.6 | **NAR** |
| 1.5 ± 0.2 | 4.6± 0.5 | 18.2 ± 0.42 | **NARNPs** |
| 2.30 ± 0.1 | 4.8 ± 0.14 | 12.45 ± 0.6 | **5-FLU** |

**Table 4**. PHD, FIH, and VHL docking scores using natural compound NAR. The protein-ligand interaction with the largest negative energy is indicated by highlighted G-Scores.

| Protein | Protein Data  Bank ID | Affinity  (Kcal/mole) | Distance (A^0^) | Docked Amino Acids | Grid dimensions |
| --- | --- | --- | --- | --- | --- |
| Prolyl hydroxylase (PHD) | **2G19** | **-10.1** | **1.3** | ARG383, TYR 303 | X=30  Y=30  Z= 30 |
| Factor inhibiting hypoxia (FIH) | **1MZF** | **-8.1** | **1.2** | HIS 279,199 | X=30  Y=50  Z=30 |
| Von Hippel Lindau (VHL) | **6BVB** | **-8.3** | **2.4** | TYR532,98  ALA510  SER111 | X=40  Y=40  Z= 40 |
